# Development and Validation of the Depression Inventory for Type 1 Diabetes (DID-1)

**DOI:** 10.3390/ijerph182312529

**Published:** 2021-11-28

**Authors:** Mónica Carreira, María Soledad Ruiz de Adana, Marta Domínguez, Sergio Valdés, Maria Cruz Almaraz, Gabriel Olveira, María Teresa Anarte

**Affiliations:** 1 Department of Personality, Assessment and Psychological Treatment, Faculty of Psychology, University of Málaga, Campus de Teatinos, 29071 Málaga, Spain; mcarreira@uma.es; 2 Instituto de Investigación Biomédica de Málaga (IBIMA), 29010 Málaga, Spain; solruizdeadana@gmail.com (M.S.R.d.A.); mmelilla@yahoo.com (M.D.); sergio.valdes@hotmail.es (S.V.); malmaraza@hotmail.com (M.C.A.); gabrielm.olveira.sspa@juntadeandalucia.es (G.O.); 3 Clinical Management Unit of Endocrinology and Nutrition, Regional University Hospital, 29010 Málaga, Spain; 4 Centro de Investigación Biomédica en Red de Diabetes y Enfermedades Metabólicas Asociadas (CIBERDEM), Instituto de Salud Carlos III, 28029 Madrid, Spain

**Keywords:** type 1 diabetes, depression, depressive symptoms, scale development, Depression Inventory for type 1 Diabetes

## Abstract

People with type 1 diabetes (T1D) are more likely to have depression than the general population and their prognosis is worse. Unfortunately, the characteristics of persons with T1D lead to inadequate screening for depression in this population. To aid in the detection of depression in this population, this study was undertaken to develop a depressive symptoms assessment instrument specific to patients with T1D and to examine its psychometric properties. A total of 207 people with T1D participated in this study. The reliability of the new scale was assessed using Cronbach’s alpha and the Spearman-Brown split-half coefficient. The Depression Inventory for type 1 Diabetes (DID-1), composed of 45 items on a Likert scale (1–7), shows high internal and temporal consistency, as well as adequate concurrent, convergent and discriminant validity. Factor analysis identified 7 factors (Symptoms of depression, Diminished interest, Hopelessness and dissatisfaction, Guilt, Fear, frustration and irritability, Defenselessness, and Interference in daily life) that explained 61.612% of the total variability. The cut-off score for diagnosis was set at 155 points. It was concluded that the DID-1 scale is a reliable, valid and useful tool for the assessment of depressive symptoms, eliminating the bias of other nonspecific diabetes scales.

## 1. Introduction

People with diabetes have a higher risk of depression than those who do not have this disease [1,2]. The presence of diabetes complications [3,4] or of other physical diseases together with the diagnosis of diabetes [5] increases the risk of experiencing depression, and the risk of diabetes complications is also more prevalent in people with depression [6]. In addition, the course of depression in individuals with diabetes is less favorable than in people with depression but no other chronic disorder [7].

The literature does not usually differentiate between type 1 diabetes (T1D) and type 2 diabetes (T2D), but it is necessary to do so, since the psychosocial factors that play an integral role in managing diabetes differ according to type. T1D and T2D are distinct conditions that have different psychological effects [8,9,10]. Furthermore, although higher depression scores have been reported in people with diabetes, the prevalence rate of depression is three times higher in people with T1D (12%, range 5.8–43.3% vs. 3.2%, range 2.7–11.4%) and almost twice as high in people with T2D (19.1%, range 6.5–33% vs. 10.7%, range 3.8–19.4%) compared to people without this disease [11]. In a study carried out in Spain, in which the Mini International Neuropsychiatric Interview (MINI) was used for assessment, the prevalence of depression was found to be 24% in people with T1D [12]. Indeed, several factors have been found to increase the risk of developing depression in T1D, such as being a woman, not being employed, smoking, having complications due to T1D or another physical condition, not perceiving support from family, friends or coworkers in relation to T1D, high number of weekly episodes of hyperglycemia and poor quality of life [13].

Therefore, given that the risk of developing T1D is not only higher but is also increasing [14,15], the need to detect depression in people with T1D is a priority, which makes the evaluation of depression in these people essential.

Although the proper evaluation of depressive disorder in patients with T1D is crucial in order to address the disease, the results are far from desirable; Anarte et al. [16] found that 25% of people with T1D and depression are not detected at their visit to the doctor. On the other hand, Poulsen et al. [17] found that, although the prevalence of depression and anxiety in their sample was high, less than half were detected by the medical team.

Recently Davis et al. [18] developed a validated combined depression-anxiety metric, the Diabetes Anxiety Depression Scale (DADS), for potential clinical application in people with T2D. However, it is not a specific instrument for measuring depression in T1D. Consequently, this study was carried out for the purpose of obtaining an accurate and specific measure of depression in T1D.

Diagnostic criteria from the Diagnostic and Statistical Manual of Mental Disorders (DSM) for depressive disorders are the gold standard for carrying out an appropriate assessment of clinical depression. Nevertheless, due to time constraints and costs involved in performing this assessment, this method is rarely used in patients with diabetes, both T1D and T2D (hereinafter the term diabetes will be used in this sense since the literature does not differentiate between them) [19]. This is why questionnaires, which are more quickly and easily completed, are often employed to assess depression in these patients. However, the results, as seen above, are not desirable. In addition, the assessment instruments currently used for this purpose do not measure the same construct [20] and present several limitations:

First, the prevalence of depression in individuals with diabetes varies systematically depending on the method used for its identification [21]. Although several studies have analyzed the psychometric properties of depression questionnaires in patients with diabetes, obtaining positive results [22,23], these studies report little data on their reliability and validity [22,24,25]. Some authors believe that generalizing the results of studies conducted solely with self-administered questionnaires carries the risk of overestimating the prevalence (high “false positive” rate) [26,27] and that a proper assessment would first require a diagnosis based on a clinical interview or using only questionnaires validated specifically for the study population [28].

Second, it is also believed [26] that self-report measures are not specific for assessing depression in these patients because high scores may be due not only to the presence of depression but also to other comorbid disorders such as anxiety, stress, or even to the diabetes itself.

Third, patients with depression may have suboptimal glycemic control, which would facilitate confusing depressive symptoms with symptoms typical of diabetes and glycemic control [29,30].

Finally, results suggest that diabetes may influence the assessment of depression [31,32], and some studies indicate that patients with diabetes who experience depression report an increase in diabetes symptoms [33]. Moreover, the evaluation system used to detect depression should take into account the life context in which depressive symptoms are occurring [34]. This last aspect raises the question of the meaning of depression in diabetes and how it might be specifically assessed within the context of the disease itself. Although much work has been done on its relevance to different aspects of depression and diabetes, little has been said about the construct itself. Snoek et al. [35] point out that depression is a heterogeneous construct defined by a range of symptoms and the severity and impairment associated with them. If the complexity of the construct itself is compounded by the characteristics of a disease such as diabetes, it makes it difficult to detect the symptoms and, therefore, depression itself.

These problems have already been identified in other constructs such as distress or quality of life, among others, generating new instruments that assess the specific impact of diabetes. These instruments help researchers and clinicians to detect and address these issues in people with diabetes.

Consequently, with the aim of assessing the construct of depression in the context of diabetes, eliminating the limitations of the existing methods, the main objective of this study was to develop a specific questionnaire to assess depressive symptoms in patients with T1D and to analyze its psychometric properties.

## 2. Materials and Methods

**Figure 1:**
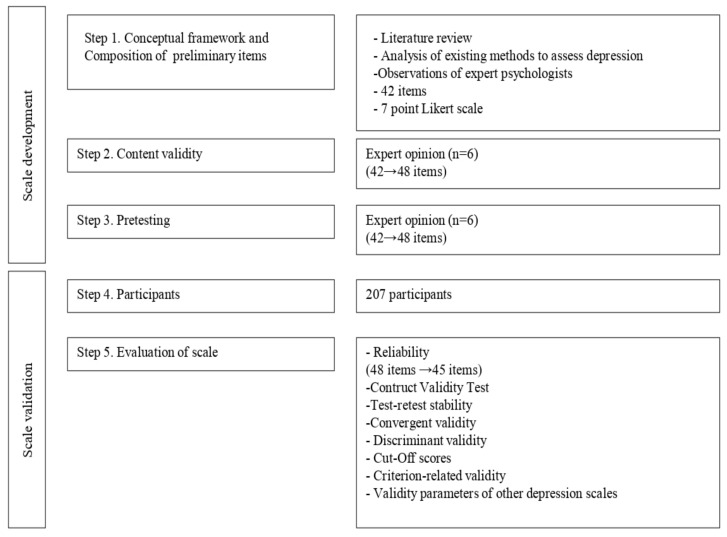
Steps of scale development and validation.

This study was conducted in two phases: scale development and scale validation (Figure 1).

### 2.1. The Depression Inventory for Type 1 Diabetes (DID-1): Scale Development

#### 2.1.1. Conceptual Framework and Composition of Preliminary Items

The Depression Inventory for type 1 Diabetes (DID-1) was developed after an analysis of the literature on the subject. In addition, we used the Structured Clinical Interview for Axis I Disorders of the DSM-IV diagnostic criteria for Major Depressive Disorder (MDD) as a reference. We also analyzed a pool of items from other depression scales, the clinical characteristics present in this population based on previous literature, and the recommendations of expert psychologists in T1D, centered on their clinical experience and observation.

Efforts were made to ensure that the items could be easily understood. The researchers reviewed all potential items to avoid redundancy. In addition, items were carefully reviewed in order to prevent any being confused with symptoms of diabetes, as stated in the previous literature and in the Introduction to this work. Finally, the initial scale was made up of 42 items and a 7-point Likert scale.

#### 2.1.2. Expert Opinion

After constructing the scale, it was given to a panel of endocrinology and nutrition specialists (*n* = 6), with broad clinical experience in a population with T1D [16], with the following instructions: (1) In your routine clinical practice, what symptoms would lead you to believe that a patient with T1D experiences depression?; (2) If any item is confusing, irrelevant, etc., please indicate the item and why it should be eliminated; (3) Comments and Suggestions. The panel contributed a series of items related to complaints made by patients with regard to their diabetes management and a major depressive episode. For example, for the symptom of loss of interest (DSM criteria for major depressive episode), clinicians referred to items such as the following: “I don’t feel like going to my medical checkups,” “I am not interested in learning anything else about diabetes”. With the suggestions given, somatic items that could have been related to T1D symptoms were eliminated (in order not to overestimate depressive symptoms), confusing items were modified and those that were relevant and repeatedly mentioned by the experts were incorporated. Thus, a scale of 48 negative items measured on a Likert scale was obtained (1. Completely disagree—7. Completely agree).

#### 2.1.3. Pretesting

Pretesting was conducted in the population of interest. Once constructed, the scale was administered to 30 patients with T1D to examine whether the items were understandable and well written. After making the appropriate changes, the scale was administered to the population that constitutes the study sample.

### 2.2. Scale Validation

#### 2.2.1. Participants and Ethics

This study included 207 patients with T1D who were seen at the Regional University Hospital of Malaga, Spain. Patients (*M* = 33.72, *SD* = 11.1 years) participated voluntarily in the study. This study was evaluated and approved by the Research and Ethics Committee of the Regional University Hospital of Malaga.

#### 2.2.2. Informed Consent

The study was first explained to the patients. Before signing the informed consent, the patients read the document and all questions were answered. All 207 patients completed the assessment. Thirty-seven patients were randomly selected for assessment with the DID-1 two weeks after the initial evaluation to study reliability.

#### 2.2.3. Measures

The demographic variables (e.g., age, gender, marital status, educational level, occupation, social support perceived, duration of diabetes, diabetes treatment) were collected through a structured interview conducted by a psychologist.

As an indicator of metabolic control, we used glycated hemoglobin (HbA1c), measured using high-performance liquid chromatography (HPLC) with a Kyoto Daiichi Kagaku device.

To diagnose the patients with T1D according to the presence of depression, the patients were evaluated with the Structured Clinical Interview for Axis I Disorders DSM-IV (SCID-I) [36] for MDD. Depression symptoms were evaluated with the Beck Depression Inventory-II and the Spanish version of the Zung Self-Rating Depression Scale. The Beck Depression Inventory-II (BDI-II) [37] evaluates the intensity of depressive symptomatology an individual has had in the last two weeks. It is a self-administered questionnaire composed of 21 multiple response items (0–3). The minimum score is 0 and the maximum 63 points. A score of 14–19 points is considered to show that the patient has symptoms of mild depression, 20–28 represents moderate depression, and 29–63 represents severe depression. In this study, we used a cut-off value of 14 (mild depressive symptoms). We obtained a Cronbach alpha coefficient of 0.94. The Zung Self-Rating Depression Scale (SDS) [38] is made up of 20 items and each item is scored on a scale of 1–4 points correlating to the frequency of behavior. The minimum score is 20 and the maximum 80 points. A score of 50–59 points is considered to show that the patient has symptoms of mild depression, 60–69 represents moderate depression, and 70–80 represents severe depression. In this study, we used a cut-off value of 50 (mild depressive symptoms). The internal consistency was *α* = 0.80. The participants were evaluated by a trained psychologist, who conducted the SCID-I interview and administered the study questionnaires.

#### 2.2.4. Data Analyses

Data analyses were performed using the Statistical Package for the Social Sciences (SPSS) version 16.0 (IBM Corp., Armonk, NY, USA) for Windows [39]. Hypothesis tests were conducted using a 95% confidence interval. To analyze the structure and construct validity of the DDI-1, item analysis and Exploratory Factor Analysis (EFA) were carried out using the Principal Components method and Promax rotation. Cronbach’s alpha coefficient and mean inter-item correlations were used to measure the internal consistency of the questionnaire. Since the number of items tends to overestimate the alpha value, the questionnaire was then randomly split into two halves, using the Spearman-Brown correlation coefficient. The temporal reliability of the DID-1 (final version) was assessed using Pearson’s correlation coefficient in a test-retest procedure two weeks after the initial evaluation. To examine convergent validity, Pearson’s correlation coefficient was used between DID-1, BDI-II and SDS. Discriminant validity was analyzed comparing the mean scores on the DID-1 using Student’s t-test between two differentiated groups: patients with and without a diabetes complication and/or disease different from diabetes. ROC (Receiver-Operating Characteristic) methodology was used to obtain a cut-off point for the DID-1 questionnaire, with the structured interview from the DSM-IV (SCID-I) as the diagnostic criterion. Concurrent validity was evaluated using Student’s t-test to determine whether significant differences indeed existed between the mean scores obtained on the DID-1 between patients diagnosed with and without depression according to the SCID-I. The predictive value of diagnostic validity was evaluated by calculating the negative and positive predictive values of the DID-1, based on the prevalence of the disorder, and comparing these results with those obtained in the same study with the BDI-II and the SDS.

## 3. Results

### 3.1. Sample Characteristics

The sample comprised 207 patients with T1D, of whom 99 were men (47.8%) and 108 women (52.2%), with a mean age of 33.72 years (range: 15–65, *SD* = 11.10). The characteristics of the sample are presented in Table 1.

The characteristics of the sample selected for the test-retest were not significantly different from those of the rest of the population for the principal sociodemographic variables: gender, age, time of diagnosis of diabetes and percentage of patients diagnosed with depression according to the structured interview. No significant differences in glycemic control were observed.

### 3.2. Evaluation of Scale

#### 3.2.1. Reliability

The initial scale, composed of 48 items, demonstrated high internal consistency, as reflected by a Cronbach’s alpha coefficient of 0.958. Three items presented low correlation with the total scale (*r* < 0.30) and were therefore eliminated. The internal consistency of the resulting scale (DID-1), composed of 45 items, was equally optimal based on Cronbach’s alpha coefficient (*α* = 0.960) and the value of the Spearman-Brown coefficient for the random split-half method (*r*_SB_ = 0.897). Hence, the mean inter-item correlation was *r* = 0.338.

#### 3.2.2. Construct Validity Test

A factor analysis was carried out with the resulting 45-item scale. In this case, the condition of seven factors was chosen.

Factor analysis suitability was made evident by the KMO index (0.919) and the high significance of the Bartlett test (*χ*^2^(990) = 5903.928, *p* < 0.001). The 7-factor solution explained 61.612% of the total variability. An item was said to associate with the factor for which it had the largest loading (Table 2). Correlations between factors were moderate (Table 3).

On the other hand, significant differences were found in the scores for the total DID-1 scale and each of the seven sub-factors depending on whether or not patients had depression according to the SCID-1 interview (Table 4).

#### 3.2.3. Test-Retest Stability

Test-retest assessment was calculated using Pearson’s correlation coefficient (*r* = 0.824). This high value indicates that the resulting DID-1, composed of 45 items, exhibits good temporal consistency.

#### 3.2.4. Convergent Validity

The scores obtained by patients on the DID-1 showed high correlation with scores obtained on other depression questionnaires: in this case the BDI-II (*r* = 0.884) and the SDS (*r* = 0.788), which shows that the convergent validity of the DID-1 is adequate. Although the correlation obtained with the SDS was slightly lower, it must be kept in mind that eight items in this scale are somatic symptoms.

#### 3.2.5. Discriminant Validity

The scores obtained on the DID-1 differed significantly between the patients who experienced a physical condition and those who did not. The mean DID-1 scores in the first group (*M* = 125.30, *SD* = 54.52, range: 45–301) were significantly higher than in the second group (*M* = 101.97, *SD* = 44.22, range: 45–248), *t* (132.078) = 3.174, *p* = 0.002. For these reasons, the discriminant validity of the DID-1 scale is adequate.

#### 3.2.6. Cut-Off Scores

Using ROC curves, a cut-off score for the DID-1 was obtained, using the DSM-IV (SCID-I) structured interview as the diagnostic criterion. Once this classification was complete, the sensitivity (Se) and the specificity (Sp) of the test were determined. Youden’s index (Se + Sp− 1) was used to measure the mean gain in certainty.

**Figure 2:**
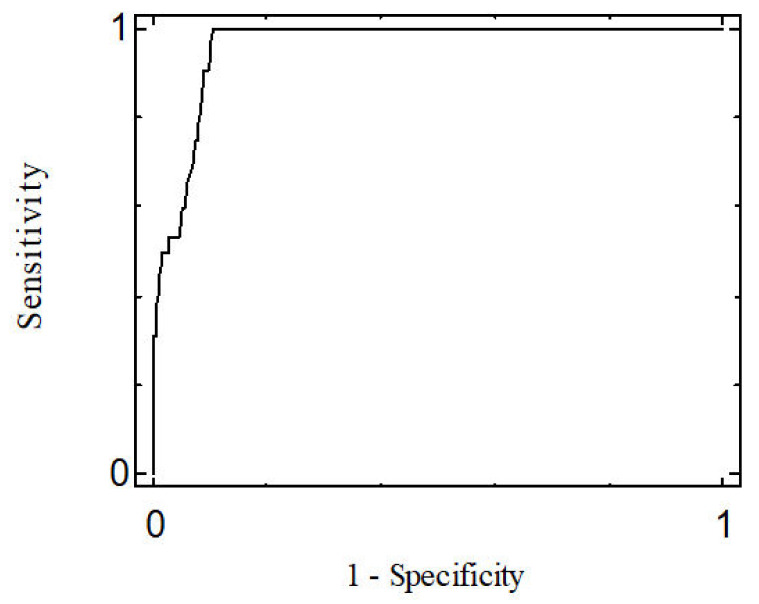
ROC Curve.

A cut-off score was set at 155, which enabled the establishment of the presence of depressive symptoms after administration of the DID-1. This cut-off score was chosen for its high sensitivity (0.906) and specificity (0.909), taking into account that both were greater than 90%. The area under the ROC curve (AUC) was 0.961 and its significance probability (*p* < 0.001) supports the hypothesis that the curve moves sufficiently away from the diagonal and thus has high discriminant power (Figure 2).

The percentage of false positives was 9.14%, while that of false negatives was 9.38%. If false positives are considered equally as serious as false negatives, a mean gain in certainty of 0.815 is obtained on calculation of the Youden’s index. Thus, the minimum score that can be obtained on the DID-1 questionnaire is 45 points and the maximum 315 points.

#### 3.2.7. Criterion-Related Validity

Concurrent validity. With regard to concurrent validity, significant differences in the total DID-1 score exist between the patients diagnosed with depression according to SCID-I (*M* = 188.47, *SD* = 33.59, range: 149–301) and those who were not (*M* = 96.29, *SD* = 37.03, range: 45–198), the scores being higher, and therefore more unfavorable, in the group of patients diagnosed with depression according to SCID-I, *t* (205) = 13.126, *p* < 0.001.

Predictive value of diagnostic validity. Taking the prevalence obtained in the study according to SCID-I (15.46%), the positive and negative predictive values of diagnostic validity of the DID-1 were the following: positive predictive value (PPV) = 0.644, and negative predictive value (NPV) = 0.981.

#### 3.2.8. Validity Parameters of Other Depression Scales

BDI-II and SDS. The results of the three depression scales used in this study can be seen in Table 5. The DID-1 is the most balanced of the three scales, as it is the only one with a sensitivity and specificity greater than 90%, displaying similar values for false positives and false negatives. It also had the best Youden’s Index, presenting only a slight difference when compared to the BDI-II, but with a marked difference compared to the SDS. Regarding the existing correlations between the three scales and HbA1c in the subjects, the DID-1 results were: *r* = 0.187, *p* = 0.007; the BDI-II *r* = 0.039, *p* = 0.582; and the SDS *r* = 0.074, *p* = 0.293. Thus, the DDI-1 is the only scale that obtains a significant correlation with HbA1c at the level of 0.01 (bilateral).

### 3.3. Prevalence of Depressive Symptoms 

In this sample, the prevalence of depressive symptoms with the DID-1 was 21.74%, whereas with the BDI-II depression questionnaire it was 28.43% and 16.43% with the SDS.

### 3.4. Differences in DID-1 and 7-Factor Scores as a Function of Subject Characteristics

With respect to the results obtained with the DID-1 and the seven factors that comprise it (Table 4), in the sex variable, differences are obtained in the total score and in the seven factors, with the score being higher in women than in men, with this difference being statistically significant in the total score (*p ≥* 0.001) and in the following factors: Factor 1 (*p ≥* 0.001), Factor 3 (*p* = 0.005), Factor 5 (*p* = 0.002) and Factor 7 (*p* = 0.043). With regard to patients with physical illness, differences were found between those with physical illness and those without, and this was significantly higher in those with physical illness in the total score (*p* = 0.002) and in the following factors: Factor 1 (*p* = 0.001), Factor 3 (*p ≥* 0.001), Factor 5 (*p* = 0.029) and Factor 7 (*p* = 0.049). No significant differences were found according to the age of the participants or the length of diabetes duration. With respect to glycemic control, significant differences in HbA1c were found in Factor 2, with higher scores in those subjects with HbA1c ≥ 7.0%.

## 4. Discussion

Depression is commonly identified in association with a number of chronic medical illnesses and is most strongly associated with diabetes mellitus [40], with the prevalence rate of depression being three times higher in people with T1D than in the general population. However, the evaluation of depressive disorder in this population has been problematic. In this regard, Fisher et al. [41] called for greater clarity and accuracy to define and measure ‘depression’ in a consistent manner.

Many of the somatic symptoms of depression (e.g., change in appetite resulting in weight gain or loss, loss of energy and difficulty concentrating) could be attributed to diabetes, making it difficult to determine a specific attribution to comorbid depression [42]. Clark et al. [43] insist that: suicidal ideation, sense of failure, sense of punishment, loss of social interest, indecision, and dissatisfaction may represent criteria for depressive severity that are not confounded by the presence of physical illness or the attendant distress. One of the most widely used instruments in clinical psychology and psychiatry to assess the severity of depression in patients with mental disorders and to detect possible depression in normal populations is the BDI. However, it has been questioned by clinicians because it includes eight somatic symptoms that may be spuriously producing elevated estimates of the prevalence of depression in patients with medical problems, as these symptoms of depression overlap with symptoms caused by many medical disorders. For example, in patients with diabetes, a somatic symptom of depression on the BDI-II, such as tiredness or fatigue, may be related to complications produced by the medical disorder, or else be an indicator of depression. For this reason, Beck et al. [44] constructed the BDI-FastScreen to reduce the number of false positives for depression in patients with medical problems. However, the BDI-FastScreen is not a diabetes-specific instrument. As Hermanns et al. [42] indicates, screening needs to be implemented in these practices, but other factors will also influence the usefulness of screening for comorbid depression. These include selecting the most sensitive and specific test, among other measures.

Recently, Davis et al. [18] developed a validated combined depression-anxiety metric, the Diabetes Anxiety Depression Scale (DADS), for potential clinical application in people with T2D; however, it is not a specific instrument. In this work, we have obtained a new specific instrument to measure depression in people with T1D that aims to reduce the number of false positives for depression in patients with T1D, by excluding overlapping somatic symptoms.

This new scale (DID-1) was developed to assess depressive symptoms in patients with T1D, using the Structured Clinical Interview for Axis I Disorders DSM-IV (SCID-I) for MDD as the gold standard. It proved to be a useful tool for the correct assessment of depression in these patients. To achieve this, our research team was scrupulous in the conceptual definition of the disorder “depression” in order to obtain results for this variable that could not be confused with the presence of other psychological disorders related to diabetes. Thus, in the process of creating the instrument, special attention was paid to the item selection phase, including those items that met DSM-IV diagnostic criteria for MDD, as well as the clinical criteria used by endocrinologists and psychologists treating patients with diabetes. The most commonly used depressive symptoms assessment scales, such as the BDI-II and the SDS, were considered, and care was taken to avoid those items that might overestimate the presence of depressive symptoms in patients with diabetes, such as change in appetite, weight gain or loss, loss of energy or difficulty in concentrating, etc.

The results show that the DID-1 has excellent psychometric properties, with high internal consistency and test-retest reliability. In addition, as was expected, the results support the convergent and discriminant validity of the new instrument. Furthermore, the scale discriminates well between those patients who present depressive symptoms and those who do not. The definitive questionnaire of 45 items is composed of seven factors that explain 61.612% of the total variability. Each of the seven factors is related to symptoms of depression and correctly discriminates between individuals with and without depression according to SCID-I. In this sense, Factor 1 items comprise the main symptoms of a major depressive episode. This Factor includes one of the two cardinal symptoms of depression according to the DSM (depressed mood). Factor 2 items refer to the second cardinal symptom of depression according to the DSM (diminished interest). This factor includes items that reflect loss of interest in self-care of diabetes, observed by psychologists and medical specialists and reported in the literature [45,46,47], as a cause of psychological impairment in the patient [48]. Factor 3 (Hopelessness and Dissatisfaction) contains one of the characteristic aspects of mood and diabetes care set out in the literature. van Tilburg et al. [49] indicate that patients with T1D would be more likely to manifest learned helplessness if they observed that their attempts to control their diabetes were not successful. In addition, we must not forget that Hopelessness and Dissatisfaction are two diagnostic criteria of MDD according to the DSM. Factor 4 (Guilt) is another symptom listed in the DSM criteria, and items refer to the guilt a patient may experience, which, in the case of diabetes, may be related to the disease (for example: “I think my diabetes is a punishment”). Factor 5 items (Fear, frustration and irritability) refer to fear of the future, anger and longing/impossibility of returning to life before diabetes (for example: “I feel like I can’t live a normal life like everyone else”). We should bear in mind that depression is more than sadness, so the mood can be anger or restlessness as indicated in the DSM. The three items comprising this factor show a moderately high correlation with the total scale. In addition, this factor shows adequate correlation with the remaining factors of the scale (Table 3). Factor 6 items (Defenselessness) address the feeling of worthlessness (DSM criterion) that has been described in the literature as an attributional style [50], characteristic of depressed individuals, as manifested by the population with diabetes in relation to their disease (for example: “I don’t believe any treatment can improve my diabetes”). Finally, Factor 7 items (Interference in daily life) refer to other depressive symptoms and the interference that diabetes has in the daily life of the patient (sleep disorders and decreased ability to think or concentrate). For example: “It is hard for me to concentrate because of my diabetes.” In fact, the degree of interference is one of the criteria for the diagnosis of MDD (Criterion B of the DSM).

In sum, the seven factors of the DID-1 are the following:

Factors 1 and 2: cardinal symptoms of depression seen in persons with T1D.

Factors 3 to 7: Other symptoms of depression included in the DSM diagnostic criteria for MDD and with theoretical support, described in persons with T1D.

Accordingly, all seven factors refer to symptoms of depression in patients with T1D, precisely differentiating between depressive disorder and other psychological disorders.

The prevalence of depression found in this study through the SCID-I is low in comparison with the estimates of some authors, who place the prevalence of depression in patients with T1D at around 30% [12,14,21]. Perhaps, for this reason, the positive predictive value (PPV) is not very high in any of the three scales, although it is high enough to assure that the DID-1 confirms the disease. Additionally, the high negative predictive value (NPV) indicates that the DDI-1 is a helpful tool for ruling out depression in patients with T1D. Therefore, in a population with a high prevalence of depression (as is the general case in patients with T1D), the DID-1 scale proves to be a useful instrument for confirming as well as for ruling out the disease.

Despite not providing the best results in terms of sensitivity and specificity, the DID-1 is the most balanced of the three scales, as it is the only one of the three scales displaying levels of greater than 90% in both validity parameters and in false positives and negatives. Moreover, this scale demonstrates the best accuracy for the identification of depressive symptoms in patients with T1D. The complexity of the disease was taken into consideration in the creation of this tool, resulting in a highly reliable instrument.

When assessing differences in DID-1 scores as a function of sample characteristics, we found that women scored worse than men on both the total scale score and the seven factors. These results are consistent with previous results and those found in the general population and in the population with diabetes, with a higher prevalence of depression in women [14,35]. With regard to complications, significant differences were found between those with physical illness and those without. This aspect has been noted in previous literature and recently reviewed for the European Depression in Diabetes Research Consortium [6]. Although previous studies have found that participant age and duration of diabetes were related to depression [14], in our study no significant differences were found in terms of the presence of depressive symptoms, perhaps due to the relatively young age of the population. With respect to glycemic control, recent publications suggest a link between depression and poor glycemic control [14,51]. In this study, we found significantly worse scores on Factor 2 (Diminished interest) in those subjects with an HbA1c greater than or equal to 7%. This result indicates that scores on the questions in this factor may help to identify poor glycemic control behaviors in the patient.

Although a protocol for screening for depression symptoms in people with T1D has not yet been created [52], previous studies have shown that systematic screening for depression in patients with diabetes increases the detection and treatment of this disorder [53]. The DID-1 is a good instrument for screening or initial assessment to determine the presence of depressive symptoms in patients with T1D.

## 5. Conclusions

The new instrument (DID-1) is a good specific tool to aid clinicians in making an accurate assessment of depressive symptoms in patients with T1D. This facilitates appropriate treatment and prevents relapses and chronification, as well as possible complications associated with the presence of depression in these patients.

The DID-1 scale is composed of 45 items evaluated on a Likert scale (1. Completely disagree—7. Completely agree) and seven factors (Symptoms of depression, Diminished interest, Hopelessness and dissatisfaction, Guilt, Fear, frustration and irritability, Defenselessness and Interference in daily life) that represent symptoms of depression present in people with T1D. To determine whether or not the subject has depression, the items must be summed. A score of 155 points or more indicates that the subject has depressive symptomatology.

The new instrument has several advantages:

- The DID-1 has excellent psychometric properties, with high internal consistency and test-retest reliability.
- The DID-1 is a depression assessment instrument designed specifically for people with T1D.
- The DID-1 is a good instrument for screening or initial assessment to determine the presence of depressive symptoms in patients with T1D.
- The DID-1 offers advantages compared with the scales used in the general population: The scores on these scales are usually distributed according to the severity of the symptoms (mild, moderate…). With this instrument we are providing healthcare professionals with a tool to detect whether or not the patient has depressive symptoms.
- The DDI-1 is also a useful tool for research in T1D, as it is specific to this population, has good psychometric properties and its use would standardize studies using the same assessment tool.

Limitations of the study:

A possible limitation of the study is that the resulting scale is rather long for application in clinical consultation. A future study will entail the development of a short version that allows its application in a fast and efficient way.

In this study, no measure of distress was administered. Given the correlation found in several studies between depression and distress, this would have been of interest. However, we were not able to include a measure of distress in this study because the Spanish translation was completed only recently by our research group and has not yet been published [54]. Further studies on this new instrument are needed to continue to improve it.

Type 1 diabetes (T1D) can influence the evaluation of depression, even in standardized clinical interviews administered by trained professionals, considered the gold standard of assessment. Measurement of depression in people with T1D must therefore be improved [31]. The new instrument developed, the DID-1, is intended to respond to this need.

## Figures and Tables

**Table 1:** Demographic characteristics of the total sample (*N* = 207).

	Depression (SCID-I) (*N* = 207)		Depression (DID-1) (*N* = 207)	
Variable	Yes (*n* = 32)	No (*n* = 175)	Yes (*n* = 45)	No (*n* = 162)
Sex				
Men	6 (18.8)	93 (53.1)	12 (26.7)	87 (53.7)
Women	26 (81.3)	82 (46.9)	33 (73.3)	75 (46.3)
Physical illness				
Yes	20 (62.5)	56 (32)	26 (57.8)	50 (30.9)
No	12 (37.5)	119 (68)	19 (42.2)	112 (69.1)
Age of the participants *	40.41 ± 10.84 (18–57)	32.60 ± 10.74 (15–65)	36.24 ± 11.68 (16–57)	33.12 ± 10.86 (15–65)
Diabetes duration *	16.82 ± 9.85 (0.07–35)	15.08 ± 9.65 (0.04–50)	15.57 ± 9.77 (0.07–36)	15.28 ± 9.64 (0.04–50)
HbA_1c_ (%) *	7.8 ± 1.8 (5.70–14)	7.6 ± 1.3 (5.10–14)	7.76 ± 1.67 (5.10–14)	7.57 ± 1.32 (5.20–14)
HbA_1c_ (mmol/mol) *	62 ± −3.82 (39–130)	60 ± −9.3 (32–130)	61 ± −5.24 (32–130)	59 ± −9.07 (33–130)

*n* (%) (Frequency and percentages); * Mean, Standard Deviation (M ± SD) and Range.

**Table 2:** Factor loading matrix after Promax rotation.

Items	Factor 1	Factor 2	Factor 3	Factor 4	Factor 5	Factor 6	Factor 7
FACTOR 1: Symptoms of depression							
2. People around me have noticed sudden changes in my mood	**0.877**	−0.114	−0.292	0.124	−0.007	0.072	0.101
3. I often have feelings of emptiness or sadness	**0.870**	−0.063	0.051	−0.039	0.113	−0.065	−0.050
1. My general mood isn’t good	**0.869**	−0.095	0.020	−0.051	0.048	−0.085	0.075
4. I frequently feel like crying	**0.765**	−0.036	0.107	0.055	0.016	−0.089	0.031
25. I feel unhappy	**0.722**	0.085	0.061	0.172	−0.109	−0.060	0.104
24. I don’t feel as happy as before	**0.709**	−0.081	0.131	−0.082	0.005	0.112	0.069
5. I frequently feel like screaming, hitting or breaking something	**0.708**	−0.005	−0.224	0.124	−0.001	0.025	0.170
13. I don’t take care of my appearance	**0.685**	0.005	−0.125	−0.140	0.152	0.153	−0.116
26. My current situation drives me to despair	**0.662**	0.025	0.225	0.123	−0.007	−0.146	0.012
20. I obsess over things	**0.661**	0.038	−0.054	−0.199	0.318	−0.057	−0.142
7. I often feel like being alone	**0.660**	0.247	0.138	−0.171	−0.228	−0.149	0.035
29. Time seems to pass more slowly	**0.660**	−0.072	−0.199	0.111	−0.207	0.303	0.185
27. It terrorizes me to think about my future	**0.653**	−0.057	−0.131	0.063	0.352	0.076	−0.050
22. It is hard for me to fall asleep when I go to bed	**0.621**	−0.001	0.021	0.064	−0.057	−0.230	0.357
18. I often feel useless	**0.610**	0.040	0.390	0.112	−0.153	−0.032	−0.119
21. I am restless and it is difficult for me to stay still	**0.573**	0.039	−0.149	0.044	0.164	−0.099	0.284
6. I am more tired than usual	**0.568**	−0.023	0.060	−0.108	0.145	−0.249	0.341
28. It is difficult for me to do the things I used to do	**0.542**	0.053	0.136	−0.067	0.032	0.017	0.276
8. I think my life is going to be a failure	**0.507**	−0.004	0.400	0.054	−0.074	0.139	−0.029
11. I feel inferior to everyone else	**0.465**	0.001	0.339	−0.001	0.161	0.026	−0.205
12. Things don’t satisfy me as much as they used to	**0.459**	0.089	0.305	−0.135	0.043	0.223	0.148
19. I feel like the people who loved me have abandoned me	**0.458**	−0.042	0.174	0.382	−0.165	0.180	−0.163
10. I don’t trust people	**0.416**	0.177	0.150	−0.220	−0.179	0.189	0.100
FACTOR 2: Diminished interest							
31. I don’t want to take my glucose test results to my doctor	−0.129	**0.804**	0.031	0.088	−0.010	−0.093	0.194
40. I don’t feel like going to my medical checkups	−0.024	**0.779**	−0.061	0.090	−0.060	0.163	0.142
45. I am not interested in learning anything else about diabetes	−0.136	**0.683**	−0.193	0.080	−0.042	0.335	0.048
42. I’ve stopped taking care of my diabetes	0.157	**0.651**	−0.275	0.177	0.155	0.085	−0.294
16. I don’t take care of my health like before	0.398	**0.595**	−0.007	−0.024	0.022	−0.103	−0.221
FACTOR 3: Hopelessness and dissatisfaction							
17. I’ve thought about taking my life	−0.094	−0.130	**0.834**	0.141	0.017	−0.165	0.037
15. I think my life serves no purpose anymore	0.180	−0.182	**0.662**	0.134	0.032	0.111	0.102
32. I feel like I can’t cope with my diabetes	−0.093	0.415	**0.467**	0.069	0.271	−0.080	0.088
14. Sexual relations don’t satisfy me	0.303	−0.031	**0.316**	−0.254	0.078	0.166	0.179
FACTOR 4: Guilt							
38. I think my diabetes is a punishment	0.009	0.083	0.087	**0.712**	−0.018	0.113	−0.035
36. I am ashamed of my diabetes	−0.167	0.255	0.025	**0.664**	−0.080	0.130	0.183
33. I feel guilty about my diabetes	−0.107	0.207	0.196	**0.613**	0.126	−0.236	0.089
23. I feel like everyone would be better off if I died	0.104	−0.111	0.487	**0.532**	−0.106	−0.024	−0.127
34. I feel like I can’t take care of myself and that I need help from others	0.207	−0.048	−0.101	**0.385**	0.326	−0.145	0.247
FACTOR 5: Fear, frustration and irritability							
41. I dread thinking about the possible complications that diabetes might cause me	0.052	0.032	−0.076	−0.025	**0.787**	0.078	0.014
30. I feel angry about my diabetes	0.038	−0.014	0.323	−0.030	**0.565**	0.111	0.132
44. I feel like I can’t live a normal life like everyone else	0.070	0.009	0.120	0.039	**0.550**	0.328	0.044
FACTOR 6: Defenselessness							
43. I don’t believe any treatment can improve my diabetes	−0.128	0.104	−0.052	0.042	0.149	**0.815**	−0.076
37. I feel like I can’t do anything to improve my diabetes	0.122	0.288	−0.069	0.005	0.151	**0.486**	0.118
FACTOR 7: Interference in daily life							
9. My sleep schedule has changed (I sleep more hours or less hours than before)	0.575	0.136	−0.129	−0.052	−0.131	−0.105	**0.577**
35. It is hard for me to concentrate because of my diabetes	−0.042	0.071	0.204	0.147	0.178	0.113	**0.515**
39. I worry about my diabetes so much that I can’t think about anything else	0.106	−0.224	0.077	0.321	0.189	0.150	**0.371**

Note: The factor loading of the items in their corresponding factor has been indicated in bold. *N* = 207.

**Table 3:** Correlation matrix between factors.

Factor	1	2	3	4	5	6	7
1	1.000	0.338	0.568	0.299	0.421	0.309	0.268
2		1.000	0.308	0.125	0.370	0.238	0.115
3			1.000	0.182	0.295	0.258	0.187
4				1.000	0.300	0.166	0.143
5					1.000	0.113	0.242
6						1.000	0.163
7							1.000

Reliability for each factor was adequate, with the following Cronbach’s alpha values: *α* = 0.95 (mean inter-item correlation = 0.49) for Factor 1 (Symptoms of depression), *α* = 0.80 (mean inter-item correlation = 0.44) for Factor 2 (Neglect of diabetes care), *α* = 0.73 (mean inter-item correlation = 0.40) for Factor 3 (Hopelessness and dissatisfaction), *α* = 0.74 (mean inter-item correlation = 0.36) for Factor 4 (Guilt), *α* = 0.73 (mean inter-item correlation = 0.47) for Factor 5 (Fear and frustration), *α* = 0.62 (mean inter-item correlation = 0.45) for Factor 6 (Defenselessness), and *α* = 0.64 (mean inter-item correlation = 0.37) for Factor 7 (Interference in daily life).

**Table 4:** Differences in the scores on the DID-1 scale and in the seven factors according to the characteristics of the subjects.

	N = 207	DID-1 Scale	Factor 1	Factor 2	Factor 3	Factor 4	Factor 5	Factor 6	Factor 7
Variable	N (%)	M ± SD t/*p*	M ± SD t/*p*	M ± SD *t/p*	M ± SD t/*p*	M ± SD t/*p*	M ± SD t/*p*	M ± SD t/*p*	M ± SD t/*p*
Sex									
Men	108 (47.8)	97.79 ± 44.51	59.53 ± 31.08	11.37 ± 6.93	2.50 ± 1.51	9.44 ± 5.74	8.72 ± 4.51	4.27 ± 2.96	1.93 ± 1.55
Women	99 (52.2)	122.22 ± 51.01 −3.679/≥ 0.001	77.87 ± 36.62 −3.866/≥ 0.001	12.58 ± 6.67 −1.279/0.202	3.29 ± 2.46 −2.813/0.005	10.80 ± 5.73 −1.704/0.090	10.92 ± 5.34 −3.193/0.002	4.34 ± 2.79 −0.175/0.861	2.41 ± 1.82 −2.039/0.043
Physical illness									
Yes	76 (36.7)	125.30 ± 54.52	80.14 ± 38.86	12.46 ± 6.26	3.76 ± 2.99	10.85 ± 6.48	10.92 ± 5.47	4.66 ± 3.03	2.50 ± 1.87
No	131 (63.3)	101.97 ± 44.22 3.174/0.002	62.70 ± 31.33 3.336/0.001	11.74 ± 7.12 0.733/0.465	2.42 ± 1.06 3.757/≥0.001	9.75 ± 5.29 1.334/0.184	9.26 ± 4.74 2.209/0.029	4.10 ± 2.75 1.337/0.183	1.99 ± 1.58 1.986/0.049
Age of the participants									
<30 years old ≥30 years old	78 (37.7) 129 (62.3)	108.55 ± 46.68 111.73 ± 51.16 −0.448/0.654	65.65 ± 31.19 71.18 ± 37.41 −1.146/0.253	12.86 ± 8.07 11.49 ± 5.89 1.304/0.195	2.48 ± 1.18 3.18 ± 2.45 −2.717/0.007	10.70±5.95 9.82 ± 5.65 1.068/0.287	10.24 ± 5.23 9.64 ± 4.97 0.824/0.411	4.51 ± 3.14 4.18 ± 2.69 0.795/0.428	2.09 ± 1.77 2.23 ± 1.67 −0.581/0.562
Diabetes duration									
<15 years with diabetes ≥15 years with diabetes	101 (48.8) 106 (51.2)	111.81 ± 48.28 109.32 ± 50.70 0.362/0.718	69.93 ± 34.90 68.31 ± 35.67 0.330/0.742	12.17 ± 7.04 11.85 ± 6.61 0.336/0.737	2.86 ± 1.91 2.97 ± 2.26 −0.378/0.706	10.39 ± 5.81 9.92 ± 5.74 0.587/0.558	9.93 ± 5.16 9.81 ± 5.00 0.169/0.866	4.36 ± 2.98 4.25 ± 2.76 0.280/0.780	2.16 ± 1.65 2.19 ± 1.77 −0.167/0.868
HbA_1c_ (%)									
<7.0% ≥7.0%	71 (34.6) 134 (65.4)	104.29 ± 47.96 113.26 ± 49.70 −1.244/0.215	65.56 ± 34.05 70.57 ± 35.54 −0.974/0.331	9.80 ± 4.05 13.23 ± 7.66 −4.189/≥0.001	2.80 ± 1.95 2.99 ± 2.18 −0.614/0.540	10.09 ± 5.70 10.06 ± 5.60 0.038/0.970	9.66 ± 4.80 9.88 ± 5.19 −0.294/0.769	4.01 ± 2.78 4.45 ± 2.90 −1.050/0.295	2.35 ± 1.68 2.06 ± 1.68 1.185/0.237
Depression (SCID-I)									
Yes	32 (15.5)	188.47 ± 33.60	126.78 ± 17.84	16.50 ± 8.17	5.87 ± 3.56	14.78 ± 8.79	14.75 ± 5.41	6.09 ± 3.56	3.68 ± 2.20
No	175 (84.5)	96.29 ± 37.02 13.126/≥ 0.001	58.55 ± 26.23 18.309/≥ 0.001	11.18 ± 6.21 3.499/0.001	2.37 ± 1.01 5.513/≥ 0.001	9.31 ± 4.57 3.436/0.002	8.97 ± 4.47 6.483/≥ 0.001	3.98 ± 2.60 3.202/0.003	1.90 ± 1.44 4.406/≥ 0.001

**Table 5:** Parameters of validity of depression scales.

	DID-1	BDI-II	SDS
Sensitivity (Se)	0.906	0.969	0.719
Specificity (Sp)	0.909	0.843	0.937
False Positives (FP)	9.14%	15.70%	6.29%
False Negatives (FN)	9.38%	3.12%	28.13%
Youden’s Index	0.815	0.812	0.622
Prevalence	21.74%	28.43%	16.43%
PPV ^a^	0.644	0.534	0.676
NPV ^b^	0.981	0.993	0.948

Note. ^a^ PPV: Positive Predictive Value; ^b^ NPV: Negative Predictive Value.

## Data Availability

The data that support the findings of this study are available from the corresponding author, MTA, upon reasonable request.

## References

[1] Chireh B., Li M., D’Arcy C. (2019). Diabetes increases the risk of depression: A systematic review, meta-analysis and estimates of population attributable fractions based on prospective studies. Prev. Med. Rep..

[2] Meurs M., Roest A.M., Wolffenbuttel B.H.R., Stolk R.P., de Jonge P., Rosmalen J.G.M. (2016). Association of Depressive and Anxiety Disorders with Diagnosed Versus Undiagnosed Diabetes: An Epidemiological Study of 90,686 Participants. Psychosom. Med..

[3] Deschênes S.S., Burns R.J., Pouwer F., Schmitz N. (2017). Diabetes Complications and Depressive Symptoms: Prospective Results from the Montreal Diabetes Health and Well-Being Study. Psychosom. Med..

[4] Bąk E., Marcisz-Dyla E., Młynarska A., Sternal D., Kadłubowska M., Marcisz C. (2020). Prevalence of Depressive Symptoms in Patients with Type 1 and 2 Diabetes Mellitus. Patient Prefer. Adherence.

[5] Ishizawa K., Babazono T., Horiba Y., Nakajima J., Takasaki K., Miura J., Sakura H., Uchigata Y. (2016). The relationship between depressive symptoms and diabetic complications in elderly patients with diabetes: Analysis using the Diabetes Study from the Center of Tokyo Women’s Medical University (DIACET). J. Diabetes Complicat..

[6] Nouwen A., Adriaanse M.C., van Dam K., Iversen M.M., Viechtbauer W., Peyrot M., Caramlau I., Kokoszka A., Kanc K., de Groot M. (2019). Longitudinal associations between depression and diabetes complications: A systematic review and meta-analysis. Diabet. Med..

[7] Holt R.I.G., de Groot M., Golden S.H. (2014). Diabetes and Depression. Curr. Diab. Rep..

[8] Browne J.L., Ventura A., Mosely K., Speight J. (2013). ‘I call it the blame and shame disease’: A qualitative study about perceptions of social stigma surrounding type 2 diabetes. BMJ Open.

[9] Browne J.L., Ventura A., Mosely K., Speight J. (2014). ‘I’m not a druggie, I’m just a diabetic’: A qualitative study of stigma from the perspective of adults with type 1 diabetes. BMJ Open.

[10] Delamater A.M., Jacobson A.M., Anderson B., Cox D., Fisher L., Lustman P., Rubin R., Wysocki T. (2001). Psychosocial therapies in diabetes. Report of the psychosocial therapies working group. Diabetes Care.

[11] Roy T., Lloyd C.E. (2012). Epidemiology of depression and diabetes: A systematic review. J. Affect. Disord..

[12] Castellano-Guerrero A.M., Guerrero R., Relimpio F., Losada F., Mangas M.A., Pumar A., Martínez-Brocca M.A. (2018). Prevalence and predictors of depression and anxiety in adult patients with type 1 diabetes in tertiary care setting. Acta Diabetol..

[13] Carreira M., Anarte M.T., de Adana M.S.R., Caballero F.F., Machado A., Domínguez-López M., Molero I.G., de Antonio I.E., Valdés S., González-Romero S. (2010). Depresión en la diabetes mellitus tipo 1 y factores asociados. Med. Clin..

[14] Aschner P., Gagliardino J.J., Ilkova H., Lavalle F., Ramachandran A., Mbanya J.C., Shestakova M., Bourhis Y., Chantelot J.M., Chan J.C. (2021). High Prevalence of Depressive Symptoms in Patients With Type 1 and Type 2 Diabetes in Developing Countries: Results From the International Diabetes Management Practices Study. Diabetes Care.

[15] Diabetes. https://www.who.int/news-room/fact-sheets/detail/diabetes.

[16] Anarte M.T., Carreira M., Ruiz de Adana M.S., Caballero F.F., Godoy A., Soriguer F.C. (2011). Precisión del diagnóstico de depresión en pacientes con diabetes mellitus tipo 1 [Accuracy of diagnosis of depression in patients with Type 1 diabetes mellitus]. Psicothema.

[17] Poulsen K.M., Pachana N.A., McDermott B.M. (2016). Health professionals’ detection of depression and anxiety in their patients with diabetes: The influence of patient, illness and psychological factors. J. Health Psychol..

[18] Davis W.A., Bruce D.G., Dragovic M., Davis T.M., Starkstein S.E. (2018). The utility of the Diabetes Anxiety Depression Scale in Type 2 diabetes mellitus: The Fremantle Diabetes Study Phase II. PLoS ONE.

[19] Fisher L., Skaff M.M., Mullan J.T., Arean P., Mohr D., Masharani U., Glasgow R., Laurencin G. (2007). Clinical Depression Versus Distress Among Patients with Type 2 Diabetes: Not just a question of semantics. Diabetes Care.

[20] González D.A., Jenkins S.R. (2014). Cross-Measure Equivalence and Communicability in the Assessment of Depression: A Focus on Factor-Based Scales. Assessment.

[21] Anderson R.J., Freedland K.E., Clouse R.E., Lustman P.J. (2001). The Prevalence of Comorbid Depression in Adults with Diabetes: A meta-analysis. Diabetes Care.

[22] Liu Y.-T., Lin L.-Y., Tuan C.-W., Yang C.-Y., Tang P.-L. (2019). Analyzing the Association HbA1c control by Depression, social participation and Utilizing Self-management Questionnaire. Diabetes Res. Clin. Pract..

[23] Rauwerda N.L., Tovote K.A., Peeters A.C., Sanderman R., Emmelkamp P.M., Schroevers M.J., Fleer J. (2018). WHO-5 and BDI-II are acceptable screening instruments for depression in people with diabetes. Diabet. Med..

[24] Roy T., Lloyd C.E., Pouwer F., Holt R.I.G., Sartorius N. (2012). Screening tools used for measuring depression among people with Type 1 and Type 2 diabetes: A systematic review: Screening tools used for measuring depression in diabetes. Diabet. Med..

[25] van Dijk S.E., Adriaanse M.C., van der Zwaan L., Bosmans J.E., van Marwijk H.W., van Tulder M.W., Terwee C.B. (2018). Measurement properties of depression questionnaires in patients with diabetes: A systematic review. Qual. Life Res..

[26] Fisher L., Hessler D.M., Polonsky W.H., Masharani U., Peters A.L., Blumer I., Strycker L.A. (2016). Prevalence of depression in Type 1 diabetes and the problem of over-diagnosis. Diabet. Med..

[27] De Joode J.W., Van Dijk S.E., Walburg F.S., Bosmans J.E., Van Marwijk H.W., de Boer M.R., Van Tulder M.W., Adriaanse M.C. (2019). Diagnostic accuracy of depression questionnaires in adult patients with diabetes: A systematic review and meta-analysis. PLoS ONE.

[28] Dantzer C., Swendsen J., Maurice-Tison S., Salamon R. (2003). Anxiety and depression in juvenile diabetes: A critical review. Clin. Psychol. Rev..

[29] Petrak F., Röhrig B., Ismail K. (2000). Depression and Diabetes. http://www.ncbi.nlm.nih.gov/books/NBK498652/.

[30] Pouwer F., Schram M.T., Iversen M.M., Nouwen A., Holt R.I.G. (2020). How 25 years of psychosocial research has contributed to a better understanding of the links between depression and diabetes. Diabet. Med..

[31] Tanenbaum M.L., Gonzalez J.S. (2012). The Influence of Diabetes on a Clinician-Rated Assessment of Depression in Adults with Type 1 Diabetes. Diabetes Educ..

[32] Van Der Donk L.J., Fleer J., Sanderman R., Emmelkamp P.M., Links T.P., Tovote K.A., Schroevers M.J. (2019). Is type of depressive symptoms associated with patient-perceived need for professional psychological care in depressed individuals with diabetes?. PLoS ONE.

[33] Ciechanowski P.S., Katon W.J., Russo J.E., Hirsch I.B. (2003). The relationship of depressive symptoms to symptom reporting, self-care and glucose control in diabetes. Gen. Hosp. Psychiatry.

[34] Gonzalez J.S., Fisher L., Polonsky W.H. (2011). Depression in Diabetes: Have We Been Missing Something Important?. Diabetes Care.

[35] Snoek F.J., Bremmer M.A., Hermanns N. (2015). Constructs of depression and distress in diabetes: Time for an appraisal. Lancet Diabetes Endocrinol..

[36] First M.B., Spitzer R.L., Gibbon M., Williams J.B.W. (1999). Entrevista Clínica Estructurada Para Los Trastornos Del Eje I Del DSM-IV (SCID-I) [Structured Clinical Interview for DSM-IV Axis I Disorders (SCID-I)].

[37] Beck A.T., Steer R.A., Brown G.K. (1996). Manual for the Beck Depression Inventory-II.

[38] Zung W.W. (1965). A SELF-RATING DEPRESSION SCALE. Arch. Gen. Psychiatry.

[39] Downloading IBM SPSS Modeler 16.0.2014. https://www.ibm.com/support/pages/downloading-ibm-spss-modeler-160.

[40] Thomas J., Jones G., Scarinci I., Brantley P. (2003). A descriptive and comparative study of the prevalence of depressive and anxiety disorders in low-income adults with type 2 diabetes and other chronic illnesses. Diabetes Care.

[41] Fisher L., Gonzalez J.S., Polonsky W.H. (2014). The confusing tale of depression and distress in patients with diabetes: A call for greater clarity and precision. Diabet. Med..

[42] Hermanns N., Caputo S., Dzida G., Khunti K., Meneghini L.F., Snoek F. (2013). Screening, evaluation and management of depression in people with diabetes in primary care. Prim. Care Diabetes.

[43] Clark D.C., Cavanaugh S.V., Gibbons R.D. (1983). The Core Symptoms of Depression in Medical and Psychiatric Patients. J. Nerv. Ment. Dis..

[44] Beck Depression Inventory^®^—FastScreen (BDI^®^-FastScreen). Pearson Assessment. https://www.pearsonclinical.co.uk/Psychology/AdultMentalHealth/AdultMentalHealth/BeckDepressionInventory-FastScreen.

[45] Egbuonu I., Trief P.M., Roe C., Weinstock R.S. (2021). Glycemic outcomes related to depression in adults with type 1 diabetes. J Health Psychol..

[46] Sartorius N. (2018). Depression and diabetes. Dialogues Clin. Neurosci..

[47] Schmitt A., McSharry J., Speight J., Holmes-Truscott E., Hendrieckx C., Skinner T., Pouwer F., Byrne M. (2021). Symptoms of depression and anxiety in adults with type 1 diabetes: Associations with self-care behaviour, glycaemia and incident complications over four years–Results from diabetes MILES–Australia. J. Affect. Disord..

[48] Alvarado-Martel D., Ruiz Fernández M., Cuadrado Vigaray M., Carrillo A., Boronat M., Expósito Montesdeoca A., Wägner A.M. (2019). Identification of Psychological Factors Associated with Adherence to Self-Care Behaviors amongst Patients with Type 1 Diabetes. J. Diabetes Res..

[49] Van Tilburg M.A., McCaskill C.C., Lane J.D., Edwards C.L., Bethel A., Feinglos M.N., Surwit R.S. (2001). Depressed Mood Is a Factor in Glycemic Control in Type 1 Diabetes. Psychosom. Med..

[50] Seligman M.E., Abramson L.Y., Semmel A., Von Baeyer C. (1979). Depressive attributional style. J. Abnorm. Psychol..

[51] Beran M., Muzambi R., Geraets A., Albertorio-Diaz J.R., Adriaanse M.C., Iversen M.M., Kokoszka A., Nefs G., Nouwen A., Pouwer F. (2021). The Bidirectional Longitudinal Association between Depressive Symptoms and HbA1c: A Systematic Review and Meta-Analysis. https://onlinelibrary.wiley.com/doi/epdf/10.1111/dme.14671.

[52] Holt R.I.G., van der Feltz-Cornelis C.M. (2012). Key concepts in screening for depression in people with diabetes. J. Affect. Disord..

[53] American Diabetes Association (2021). 5. Facilitating Behavior Change and Well-being to Improve Health Outcomes: Standards of Medical Care in Diabetes. Diabetes Care.

[54] Anarte M.T., Carreira M. (2019). Traducción al Español del Cuestionario de Áreas Problemáticas en Diabetes (Problem Areas in Diabetes-PAID).

